# Enhanced vegetable production in hydroponic systems using decontamination of closed circulating fluid

**DOI:** 10.1038/s41598-023-50974-9

**Published:** 2024-01-05

**Authors:** Shirly Lara Perez, Rafael Basilio Ferro, Bruna Corrêa, Rene Casarin, Thaila Quatrini Corrêa, Kate Cristina Blanco, Vanderlei Salvador Bagnato

**Affiliations:** 1 Universidade Federal de São Carlos, São Carlos, Brazil; 2 Instituto de Física de São Carlos, Universidade de São Paulo, Caixa Postal 369, São Carlos, 13566-970 Brazil; 3 Department of Biomedical Engineering, Texas A&M University, College Station, Texas USA; 4 IFSC/USP Avenida Trabalhador, São-Carlense, 400, São Carlos, SP CEP 13566-590 Brazil

**Keywords:** Biotechnology, Microbiology, Plant sciences

## Abstract

While plant microorganisms can promote plants by producing natural antibiotics, they can also be vectors for disease transmission. Contamination from plant management practices and the surrounding environment can adversely affect plants, leading to infections and hindered growth due to microbial competition for nutrients. The recirculation of nutrient-rich fluids can facilitate the transport of microorganisms between vegetables in the hydroponic production system. This issue can be addressed through the application of the decontamination method in the hydroponic liquid. Ultraviolet light (UV-C) has been employed for microbiology, and its effects on lettuce were evaluated in this study. This study aims to assess the effectiveness of a decontamination system using UV-C in hydroponic solutions during nutrient recirculation in hydroponics. We evaluated the time required for lettuce plants to reach their maximum height, as well as their pigment content, phenolic compounds, antioxidant capacity, and micro and macronutrient levels. The evaluation was conducted under two photoperiods (18 and 20 hours) in lettuce samples exposed to UV-C in the hydroponic fluid, with control groups not exposed to UV-C. The application of the UV-C decontamination system in hydroponic circulation water containing nutrients accelerated plant growth while maintaining nutritional values equal to or higher than those in the control groups without such a system. The results of microorganism control highlight the potential application of this technique for enhancing and expediting vegetable production. This approach reduces production time and enhances nutrient absorption and the content of certain compounds and minerals.

## Introduction

Controlled Environment Agriculture (CEA) employs precise control over environmental factors, including temperature, humidity, light intensity and photoperiod, pH, and nutrient solution concentration for hydroponic crops. Soilless crops were initially developed to mitigate soil-borne diseases. However, new diseases specific to soilless systems have emerged. While hydroponics enables plant growth without soil by providing well-balanced and safe nutrient solutions, it also introduces contamination risks to the nutrient solution through the growing substrate (root support)^1^.

Numerous microorganisms, including pathogenic bacteria, exhibit accelerated growth when exposed to high water activity levels^2^. Furthermore, certain microorganisms adapted to varying temperatures, even cold conditions, can thrive in adverse agricultural climates^3^. Elevated non-pathogenic microbial population load also intensifies competition for nutrients, ultimately diminishing the nutritional quality and accessibility of plants. Consequently, water quality plays a pivotal role in shaping the development of both roots and leaves. The substrate type and height represent crucial factors for mitigating productivity loss^4^.

Hydroponic cultivation demands meticulous attention to plant health, particularly concerning rooted systems. The high density, genetic uniformity, limited biological diversity, and continuous circulation of the nutrient solution create favorable conditions for pathogens proliferation and the emergence of epidemics. This often compels growers to turn to chemical pesticides including fungicides and bactericides, or, in extreme cases, discard the entire production^5^. In hydroponics, regular water changes are typically performed to maintain water quality and ensure the absence of harmful microorganisms in the nutrient solution. Nevertheless, there is a growing interest in hydroponics without water change, as a technique that aims to enhance vegetable production while minimizing environmental resource depletion. To the best of our knowledge, cultivars of all vegetables and flowers produced in hydroponic systems in Brazil, Canada, the USA, and France, are vulnerable to moderate or severe Pythium root rot epidemics. *Pythium spp*-induced root rot is a pervasive and frequently destructive issue across various plant species cultivated within hydroponic systems, including lettuce, arugula, cucumber, tomato, bell pepper, and spinach^6^. Given that these plants share a common nutrient solution, waterborne diseases can rapidly propagate from one plant to another, ultimately the entire crop^5,7^. The health of the roots plays a crucial role in enhancing nutrient absorption, promoting robust crop development throughout the growth cycle, and extending shelf life^8^.

Ultraviolet irradiation (UV-C) has been used to decontaminate the surface of food products and can also be implemented as part of a circulating water system to deactivate a substantial number of microbes^9–11^. UV-C light is absorbed by protein and nucleic, particularly pyrimidine and pyrimidine, leading to damage and reconfiguration of cellular components, often resulting in cellular rupture^12^. Consequently, the primary objective of this study was to decontaminate the hydroponic solution and evaluate the optimization of the vegetable production process.

## Materials and methods

### Samples

Palletized crispy lettuce seedlings of the "*Veneranda*" (*Lactuca sativa var. crispa*) were purchased from a vegetable seedling producer registered with the National Registry of Seeds and Seedlings (RENASEM SP-16678/2018). The seeds were sown on a tray with 200 polypropylene cells (16 mL/cell) filled with a coconut fiber substrate and fertilized by fertigation. Lettuce seedlings aged 37 days were used. These initial period conditions (37d) were set to allow observing the effect of variations in adult plants, the seedlings were grown in an open field and with sunlight (initial period introduced in an internal growth chamber) to be transplanted under a controlled environment until the final harvest period, which was calculated for each group until a maximum height was reached. The experimental research with lettuce was followed under Law no 10.711, OF AUGUST 5, 2003, which provides for the National System of Seeds and Seedlings and other provisions. Decree No. 5.153/2004. CHAPTER 1.

### Experimental setup

**Figure 1:**
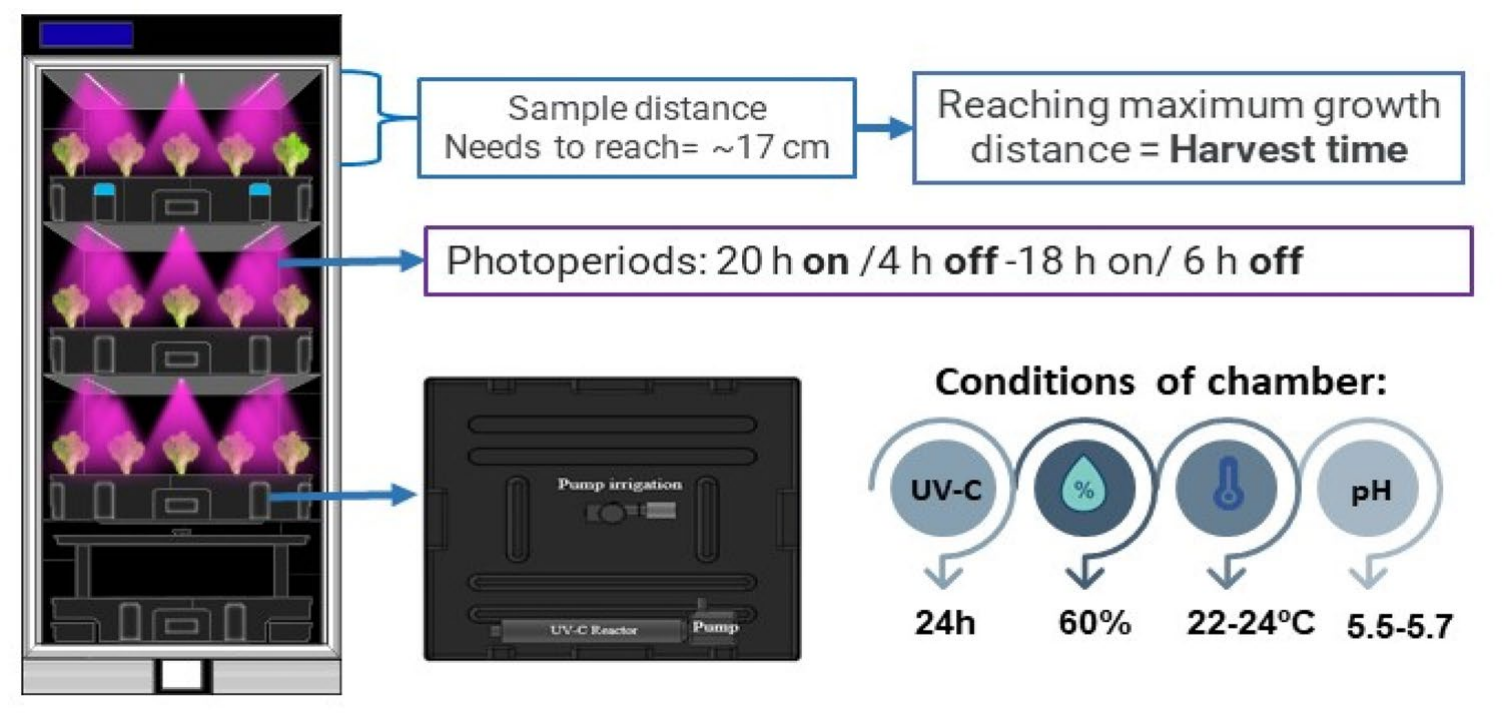
Prototype and scheme conditions for the production of lettuce with LED light using a UV-C decontamination system in the reservoir of the hydroponic solution and an irrigator pump.

The experiments of crispy lettuce were carried out in a growth chamber with controlled climatic conditions such as temperature and humidity, which were 22–24 ºC and 60% respectively, and illumination as shown in Fig. 1. The collection time was established according to the maximum height distance to be reached; therefore, the collection time was the time necessary for samples to reach 17 cm, which was the available growth space (distance between the support and the top illumination).

Different photoperiod times of 20 h of light/4 h of dark and 18 h of light/6 h of dark were tested. The nutrient solution was maintained at 2 μS/cm for electrical conductivity equivalent to environmental temperature and 5.5–6.3 pH. The nutrient solution used in this study is composed of 7.30% w/w Nitrogen (N), 12.62% w/w Potassium (K_2_O), 3.97% w/w Calcium (Ca), 2.39% w/w Sulfur (S), 0.01% w/w Boron (B), 0,01% w/w Copper (Cu), 0.05% w/w Iron (Fe), 0.01% w/w Manganese (Mn), 0.06% w/w Zinc (Zn), 0.05% w/w Nickel (Ni), 1.80% w/w Magnesium (Mg), 7.19% w/w Phosphorus (P_2_O_5_) (Plenan Ferti®) and iron was supplemented at 6% w/w (Biolchim®).

Then, the lettuce development was evaluated by a hydroponic water decontamination system containing a UV-C light reactor constantly turned on. A control group that not received UV-C light was also evaluated.

### Device and UV-C decontamination system

The UV-C decontamination system is composed of a UV-C lamp (SUNSUN-CUV305) and a pump that circulates the water placed on the tray containing the hydroponic solution. The characteristics of the system included a 5 W UV-C light power, 400 L h^−1^ reactor decontamination capacity, 400 L h^−1^ Pump Flow, and 2.0 mca Pump Pressure. Figure 1 shows the camera prototype. The first arrow is the distance that the samples (lettuce) needed to reach and the time it took, this would be the moment of harvest. The second arrow shows the photoperiods applied to the samples, which were 20 h of light from LED boards turned on (ON) and 18 h of dark (OFF). The third arrow shows the internal distribution of the tank with the hydroponic solution that was pumped to the samples, as well as the environmental growth conditions. The light dose applied was 6.12 mJ cm^−2^ and the luminous intensity was 90 μmol m^−2^.

### Leaf area measurements

The leaf area of the samples was measured by digital photography, which provided the total area followed by “Easy Leaf Area: Automated digital image analysis for rapid and accurate measurement of leaf area”^13^ and the site was analyzed by the free Software ImageJ 1.53 K (National institutes of health, USA), n = 3.

### Photosynthetic pigment content

Pigment content such as chlorophyll a, b, and carotenoid was measured colorimetrically according to Wellburn^14^. The amount of 0.1 g fresh levels dipped in 10 mL Dimethyl Sulfoxide (DMSO) was incubated for 24 h at 70ºC. The extract liquor absorbance was determined by a Varian Cary® 50 UV–Vis Spectrophotometer at 669 nm (OD_669_), 645 nm (OD_645_), and 480 nm (OD_480_).

The pigment content was calculated as follows:

$$\text{Chl a}=\left(\mathrm{12,19}*{A}_{665}\right)-\mathrm{3,45}*{A}_{649}$$

$$\text{Chl~ b}=\left(\mathrm{21,99}*{A}_{649}\right)-\mathrm{5,32}*{A}_{665}$$

$$\text{Total Car} =\frac{1000*{A}_{480}-\mathrm{2,14}Chla-\mathrm{70,16}Chlb}{220}$$

### Total phenolic compost

The extract was prepared with 250 mg of lyophilized sample diluted in 10 mL of MeOH/water (70:30 v/v) centrifuged at 4400 rpm for 20 min and filtered^15^.

Aliquot of 1 mL of the sample was placed in a test tube and diluted with 10 mL of distilled water. Then, 1.5 mL Folin Ciocalteu's (Sigma) reagent was added and incubated at room temperature for 5 min. 4 mL of 20% (w/w) Na_2_CO_3_ were added, adjusted with distilled water up to 35 mL, agitated, and left for 30 min at room temperature. The sample absorbance was measured at 765 nm against a blank, i.e., distilled water^16^.

### Antioxidant capacity by DPPH assay

The extract was prepared with 0.050 mg of lyophilized sample diluted in 20 mL methanol/water (50% v/v), placed at 80 °C (water) for 1 min, stirred for 1 h, and then filtered^15^. The free radical scavenging activity of all extracts was assessed using 1,1-diphenyl-2-picryl-hydroxyl (DPPH). A solution of 0.1 mM DPPH in methanol was prepared, and 1 mL of this solution was added to 3 mL of each extract solution. The mixtures were shaken vigorously and held at room temperature for 30 min. The absorbance was then measured at 517 nm by a UV–Vis spectrophotometer^17^.

$$\text{dpph radical scavenging activity }\left(\%\right):\left[1- \frac{{A}_{1}}{{A}_{0}}\right]\times 100$$

where A_1_ = absorbance of the sample, A_0_ = methanol solution + DPPH.

### Minerals

The protocols followed in this study were developed by Malavolta et al*.*^18^ Different techniques were used for different minerals:

- Atomic absorption spectrophotometry for Sodium (Na), Zinc (Zn), Manganese (Mn), Iron (Fe), Calcium (Ca), Magnesium (Mg), and Potassium (K).
- Colorimetry of aluminum: Aluminum (Al)
- Silver nitrate titrimetry: Chlorine (Cl)
- Metavanadate colorimetry: Phosphorus (P)
- Barium sulfate turbidimetry: Sulfur (S)
- Semi-micro-Kjeldahl: Nitrogen (N)

### Statistical analysis

The results were checked regarding their parametric distribution, and the ANOVA OneWay test was applied for data with normal parametric distribution. The significance level was 5% (*p* < 0.05), and the results are presented by mean and standard deviation.

### Statement of compliance

The authors confirm that the experimental research, including the collection of samples performed in this study, complies with relevant institutional, national, and international guidelines and legislation.

## Results

**Figure 2:**
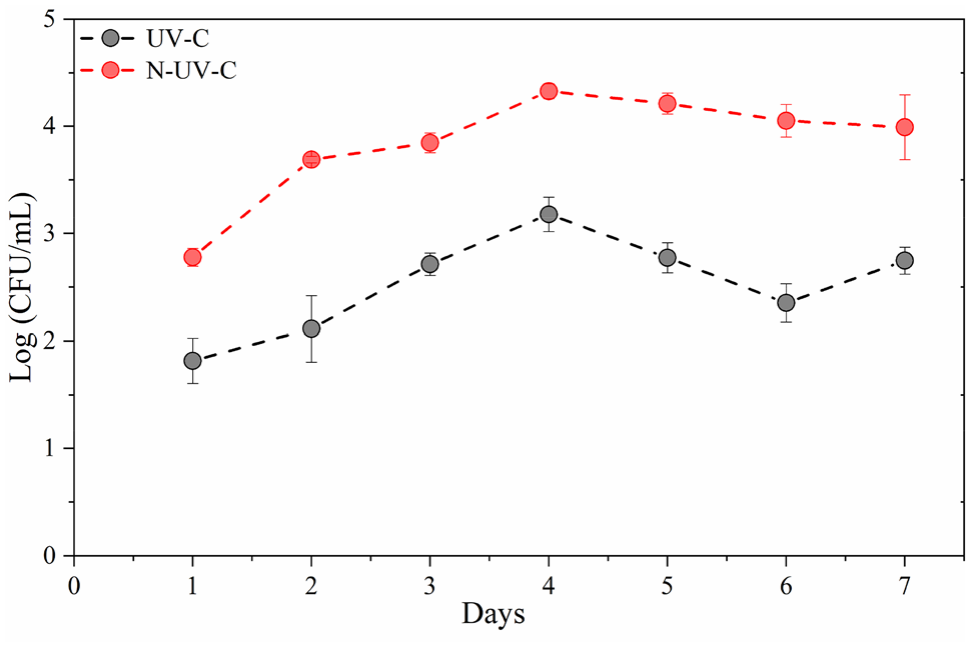
Growth curve of mesophilic bacteria with and without (N-UV-C) UV-C light in hydroponic fluid in lettuce production in an indoor hydroponic system.

The microbial quantification curve (Fig. 2) was constructed at 24 h intervals throughout 168 h, revealing consistent growth patterns and proliferation trends, whether UV-C light was applied concurrently with or independently from vegetable production. During the initial phase of the microbial growth curve (exponential phase), a significant reduction in slope was observed when UV-C light was employed. This reduction contrasted with the rapid bacterial proliferation observed in the absence of UV-C light (N-UV-C) during the initial two days of the curve. The microbial population reached its maximum levels (2.9 and 4.5 CFU/mL) when exposed and not exposed to UV-C light, respectively. This phenomenon can be ascribed to limiting factors affecting growth. However, in the stationary phase, observed in the curve without UV-C light, there was an interval between days 4 and 7, with some cells multiplying (7 days) while others perished at a similar (between days 4 and 6). Such dynamics were not observed in the UV-C light curve, where the rate of cell death exceeded that of proliferation. The UV-C-treated curve displayed a shortened microbial death phase by three days, signifying an irreversible loss of cell division capacity in an environment maintaining specific levels of salts, amount of water, and temperature favorable to microbial growth. The data imply that exposure to UV-C light harmed microorganism growth and survival, resulting in a decrease in the exponential phase, a restriction in maximum population size, and a shorter death phase, all of which are significant in microorganism control applications^19^.

**Figure 3:**
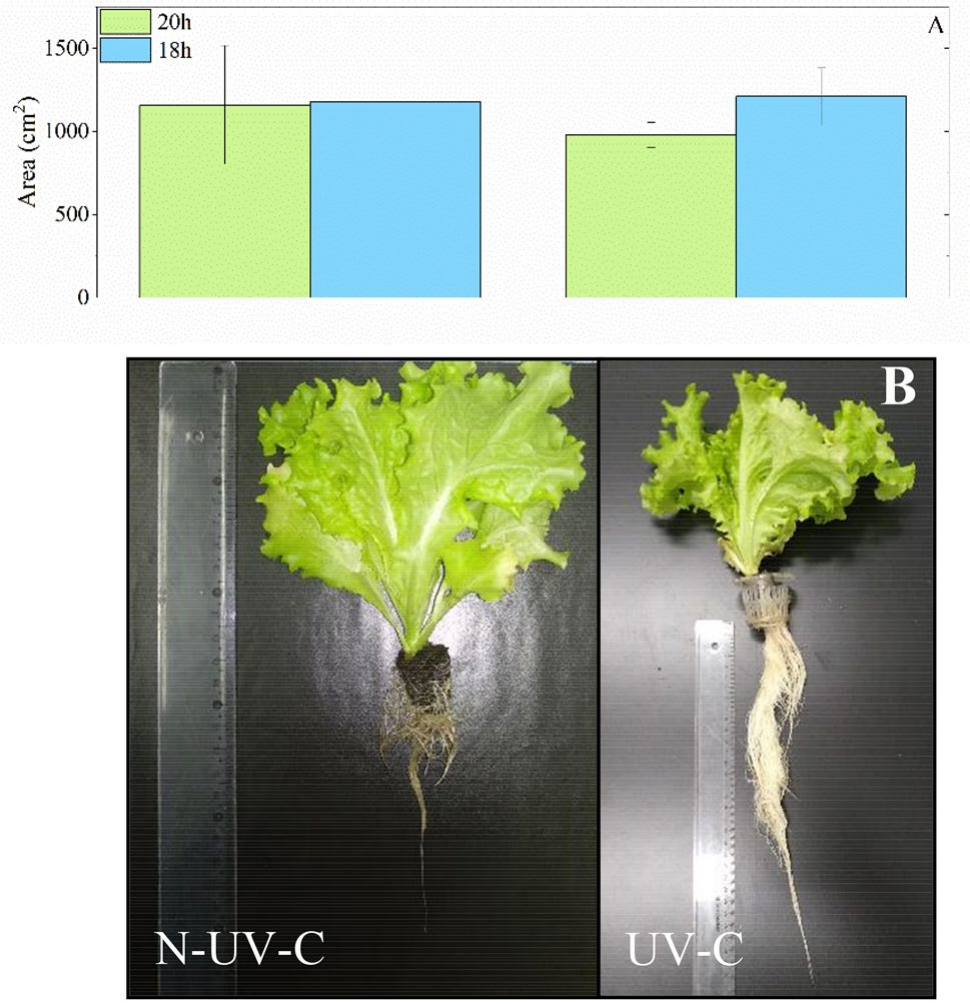
(**A**) Leaf area (cm^2^) affected by photoperiod and water decontamination of hydroponic solution in lettuce. Results are shown by mean ± standard error. (**B**) Development of roots affected by the use of decontamination UV-C system and without UV-C system (N-UV-C) in the hydroponic solution for a 20 h photoperiod.

Harvesting was done when the time required for each group to reach a height of ~ 17 cm (maximum in the growth chamber) arrived to evaluate the impact of the UV-C decontamination system. The group with a 20-h photoperiod, which reached this level in 30 days with the UV-C decontamination system (20 h-UV-C), as opposed to the 38 days required for the group without the UV-C system (20 h-NUV-C). In the case of the 18-h photoperiod experiment, the group with the UV-C decontamination system (18-h-UV-C) took 28 days to reach the target height, while the group without the system (18-h-NUV-C) required 48 days. This is an important result, showing at harvest time the importance of the microbiological quality of the hydroponic solution. The results presented in Fig. 3A demonstrate that the decontamination system significantly accelerated plant growth in comparison to the group that did not receive UV-C light. This acceleration amounted to at least 20 days for an 18 h photoperiod and eight days for a 20 h photoperiod. The reduction in the time required for growth can be attributed to intrinsic factors in plant development with microbial reduction. The analysis of total leaf area (Fig. 3A) revealed no statistically significant differences among the groups. Specifically, for the 20 h photoperiod, the leaf area C values were 1159.81 ± 356.80 cm^2^ with UV-C light and 979.32 ± 74.65 cm^2^ without UV-C light. The group subjected to the 20 h photoperiod with UV-C exhibited the highest leaf area. In contrast, for the 18 h photoperiod, the leaf area values were 1210.56 ± 172.19 cm^2^ for without UV-C light (N-UV-C) and 1177.24 ± 58.02 cm^2^ for UV-C light. These results, as well as the literature, indicate that exposure to UV-C can stimulate plant growth, possibly due to reduced microbial load in a more favorable environment ^20^. The results presented in Fig. 3B demonstrate that the implementation of the UV-C decontamination system resulted in enhanced root development, encompassing aspects of growth, cleanliness, and the number of root threads. It is important to highlight that the samples that underwent UV-C decontamination in the hydroponic fluid did not have significant changes in the growth rates of the aerial parts of the plants. This indirectly indicates the absence of significant stress effects of this procedure on plants.

**Figure 4:**
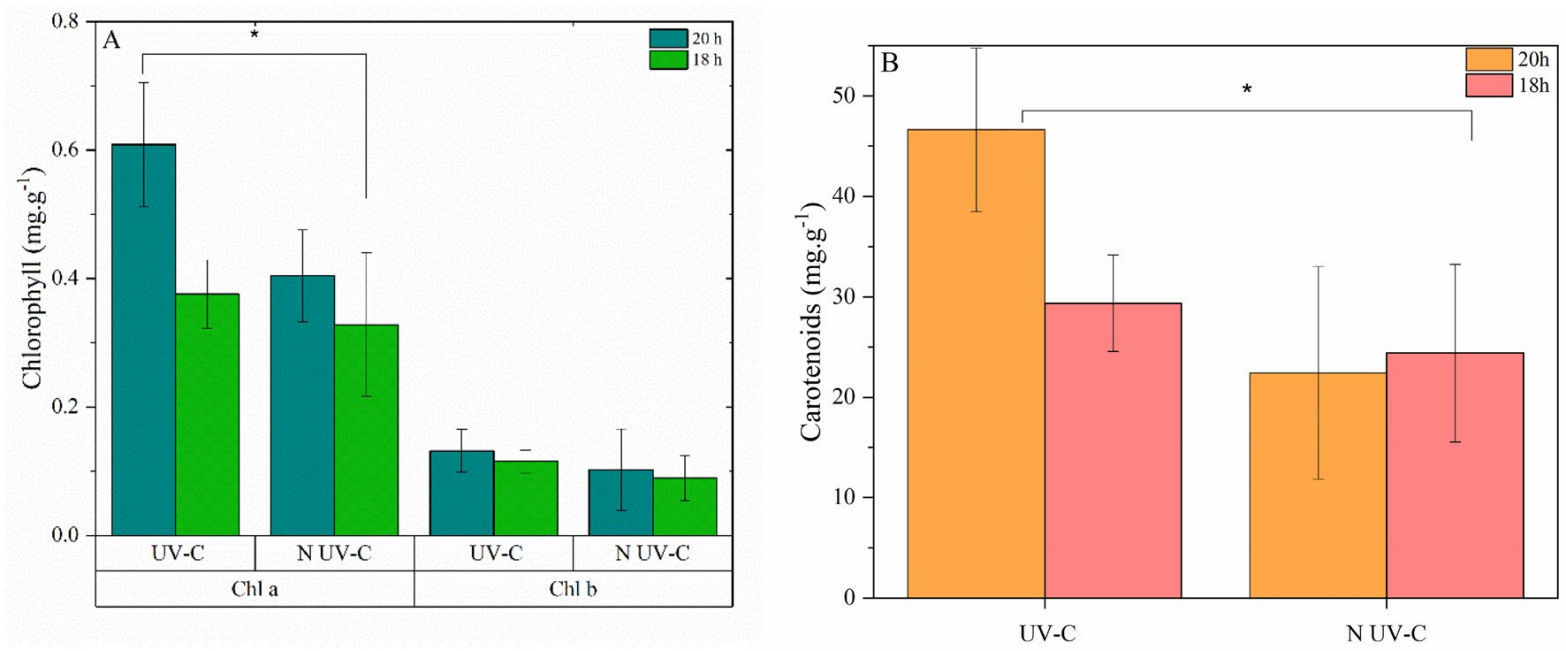
(**A**) Chlorophyll levels affected by photoperiod and water decontamination of hydroponic solution. (**B**) Carotenoid groups affected by photoperiod and water decontamination of hydroponic solution. Results are shown by mean ± standard error. (*) indicates a significant difference (*P* ≤ 0.05, Tukey’s test). Origin pro-2016 determined significant differences among the treatments for ANOVA.

The results presented in Fig. 4A indicate that the most effective stimulation of chlorophyll occurred in the group with the UV-C decontamination system and 20 h photoperiod when compared to the 18 h UV-C group. The respective values obtained were 0.61 ± 0.03 mg g^−1^ and 0.38 ± 0.05 mg g^−1^, while the photoperiods without UV-C decontamination resulted in values of 0.40 ± 0.07 mg g^−1^ for 20 h and 0.33 ± 0.11 mg g^−1^ for 18 h. Concerning chlorophyll b, there were no statistically significant differences observed among the conditions. However, the highest value, namely 0.13 ± 0.03 mg g^−1^, was recorded for the 20 h UV-C group, while the lowest value, 0.09 ± 0.04 mg g^−1^, was reported for the 18 h group without UV-C. These results suggest that exposure to the UV-C decontamination system, especially under a 20 h photoperiod, promoted a higher concentration of chlorophyll in plants^21^. This is important, as chlorophyll plays a crucial role in photosynthesis, being responsible for absorbing light and converting it into energy^22^. The increase in chlorophyll a concentration indicates a potential increase in the photosynthetic efficiency of plants exposed to UV-C light. On the other hand, chlorophyll b was not significantly affected, suggesting that it may not respond in the same way as chlorophyll a to UV-C light exposure. Therefore, the UV-C decontamination system can be an effective strategy to improve the photosynthetic efficiency of plants.

According to Fig. 4B, all carotenoids’ values were significantly different from the 20 h UV-C group, whose content was 46.64 ± 8.14 mg g^−1^.

**Figure 5:**
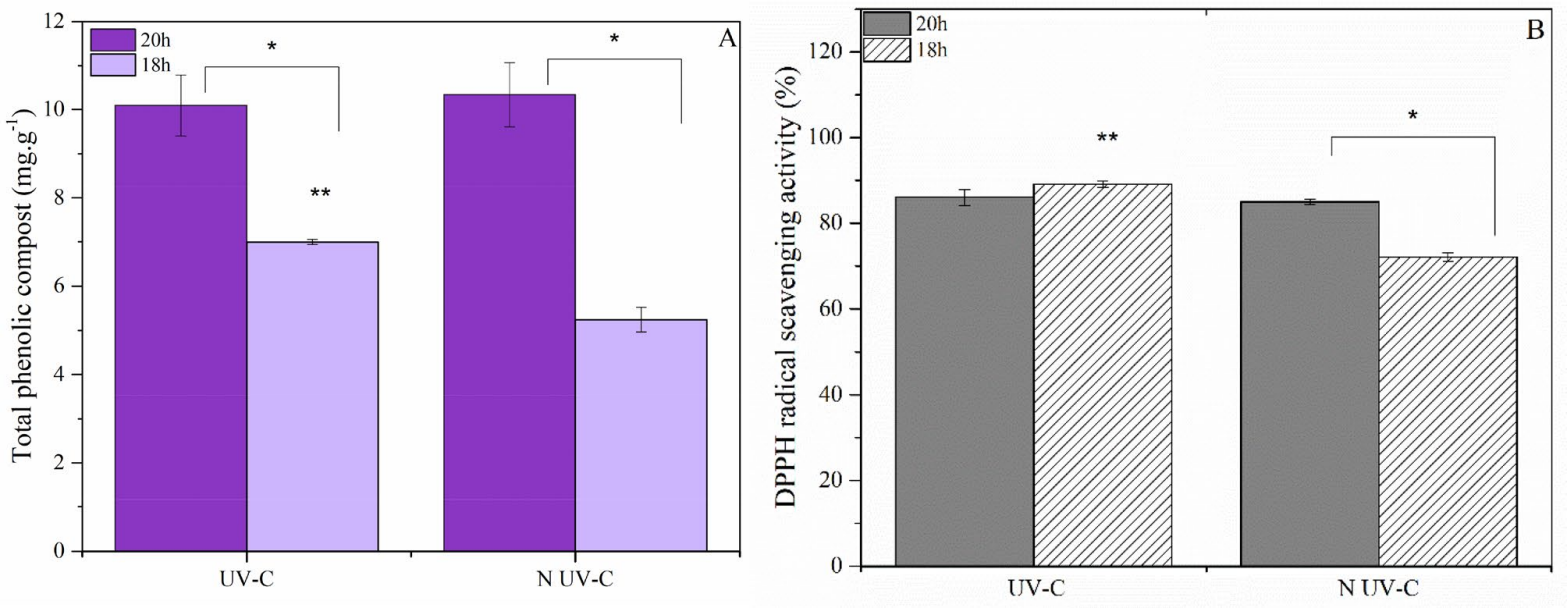
(**A**) Level of total phenolic compost affected by photoperiod and decontamination of hydroponic solution. (**B**) Inhibitory activity of DPPH radical (%) affected by photoperiod and decontamination of hydroponic solution. Data are shown by mean ± standard error. (*) indicates a significant difference and (**) denotes a significant difference with all data (*P* ≤ 0.05, Tukey's test). Origin pro-2016 determined significant differences between treatments for ANOVA.

The highest content of total phenolic compounds (10.34 ± 0.73 mg g^−1^) was observed in the 20 h N-UV-C (Fig. 5A). The 20 h UV-C group displayed values of 10.10 ± 0.69 mg g^−1^, with no statistically significant difference. For the 18 h groups, lettuces that underwent hydroponic solution decontamination exhibited a higher content of total phenolic compounds, with a value of 6.99 ± 0.06 mg g^−1^. Regarding antioxidant capacity (DPPH), the systems that employed hydroponic solution decontamination demonstrated higher values. The highest value of 89.10 ± 0.70% was reported for 18 h UV-C followed by the value of 86.10 ± 1.20% for 20 h UV-C. Only the 20 h UV-C/20 h N-UV-C groups did not exhibit a statistical difference (Fig. 5B).

**Figure 6:**
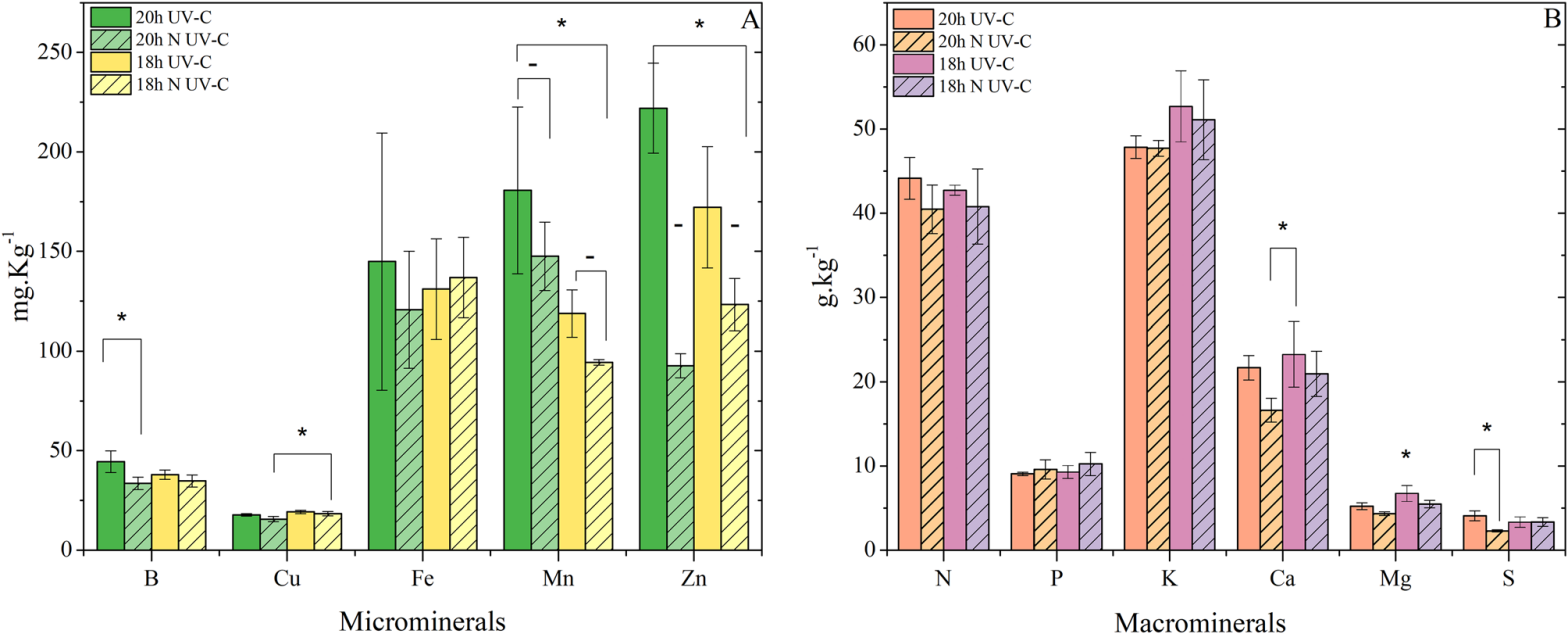
(**A**) Microminerals affected by photoperiod and decontamination with hydroponic solution water. (**B**) Macro minerals are affected by photoperiod and decontamination with hydroponic solution water. Results are shown by mean ± standard error. (*) indicates a significant difference (*P* ≤ 0.05, Tukey's test); (–) the mark in the same group does not indicate a significant difference. Origin pro-2016 determined significant differences between treatments for ANOVA.

Macro and microminerals are presented in Fig. 6A,B and according to the results, the UV-C decontamination system did not have an impact on nutrient absorption. The results indicate that UV-C treatment, especially under a 20 h photoperiod, positively affected carotenoid levels, antioxidant capacity, and the concentration of total phenolic compounds in plants, without impairing the absorption of essential minerals^23^. This suggests that the use of UV-C light can be an effective strategy to increase the nutritional and antioxidant quality of plants grown in hydroponics^24^.

## Discussion

The microbiological quality of the nutrient solution plays a vital role in vegetables growth, as bacteria and fungi can vie for nutrient uptake, thereby reducing nutrients in plants. Furthermore, certain diseases can propagate within such a nutrient solution, posing a significant challenge in hydroponic systems. The reduction of bacteria within the hydroponic solution offers potential advantages for vegetable growth by mitigating the accumulation of metabolites resulting from microbial proliferation in such environments^25^. An overabundance of these metabolites can induce various adverse effects on plant development, including impaired nutrient absorption due to heightened acidity stemming from microbial organic acids; root system obstruction caused by exopolysaccharides; stem shortening due to ethylene excess; inhibition of beneficial bacterial growth by toxic and antibiotic metabolites; and limited iron availability for vegetable development due to microbial siderophore production. These outcomes, along with established scientific literature, suggest that UV-C exposure can stimulate plant growth, potentially due to a reduced microbial load within a more conducive environment^20,26^.

The total leaf area, on the other hand, may not be significantly affected by UV-C treatment unless there are significant variations in photoperiod or other environmental factors. The UV-C system accelerated growth, resulting in taller plants in less time^27^. Furthermore, it improved root development, making them more efficient in absorbing nutrients and water absorption^28^. These results are in line with the literature, which shows that exposure to UV-C light can stimulate plant growth and reduce the microbial load in the reticular system**.**

Photosynthesis occurs with the conversion of light energy into chemical energy due to the production of carbohydrates from carbon dioxide and water with the help of chlorophyll (the green pigment of plants) in light energy^29^. Chlorophyll allows plants to capture the energy required for tissue development, and a low chlorophyll density can result in nitrogen deficiency in plant leaves, given the close connection between nitrogen and photosynthetic activity^30^. Bacterial reduction of nitrogen, an essential nutrient for organism growth^31^, can lead to a decrease in chlorophyll molecules, consequently affecting photosynthetic activity and biomass production^30^. Chlorophyll a values of 0.38 ± 0.02 mg g^−1^ and chlorophyll b values of 0.14 ± 0.08 mg g^−1^^32,33^ were reported for crisp lettuce grown with light intensity of 150 μmol cm s^−1^. The same values were achieved in this study despite the lower light intensity used. Another study reported the production of crisp lettuce through a hydroponic system, with values of 0.10–0.20 mg g^−1^ chlorophyll content^34^, which are also similar—or higher when the UV-C decontamination system was used—to the present ones.

Carotenoids are important photosynthetic pigments, since they absorb different wavelengths from chlorophylls, serving as an accessory for photosynthesis. They also protect the cells, avoiding the excess energy of excited chlorophylls reactive as antioxidants^35,36^. The more significant light stimulus observed from 20 to 18 h may have interfered with the production of those pigments, since they usually absorb light in the spectral region of the sun of maximum irradiation^37^. The contents of carotenoids obtained in this study were highest at 20 h UV-C, followed by 18 h UV-C.

Such values are high in comparison to those from the literature—in controlled environments with light intensity of 200 µmol m^−2^ s^−1^, the total carotenoid content was 0.20 ± 0.0048 mg g^−1^^38^. Other contents were found in cultures ranging from 1810 to 2760 μg/100 g dry weight in hydroponically grown samples^39^, showing the carotenoid pigment under similar conditions for lettuce samples is higher when a UV-C system is used for microbiological control.

The content of total phenolic compounds for the 18 h groups was better for lettuces that decontaminated the hydroponic solution with values of 6.99 ± 0.06 mg g^−1^, which were similar to those of iceberg lettuce purchased from a local supermarket in Colorado, USA^40^.

The mineral analysis revealed most samples achieved better results when the UV-C decontamination system was used; the difference was not significant when the result was not good, demonstrating the UV-C system did not degrade or impede the absorption of minerals in the plants. However, microminerals Mn and Zn are essential for the nutrition of bacteria and, when unavailable, proteins are used as an alternative^41^.

This study has illustrated that the uptake of manganese (Mn) and zinc (Zn) by plants can be significantly enhanced through the application of UV-C. This enhancement primarily results from the competition between plants and bacteria for nutrients. Consequently, a reduced bacterial population in hydroponic production leads to increased nutrient availability for the plants. Typically, the zinc content in lettuce falls within the range of 30 to 46 mg g^−1^ of dry weight. However, the values obtained in this study notably surpass those reported in existing literature, with the 20 h UV-C light group recording 221.30 ± 22.70 mg g^−1^, as previously documented. Literature reports suggest similar or even higher values for various minerals of interest, such as potassium (K), which usually varies from 2.35 to 6.47 mg kg^−1^ under different conditions for various lettuce cultivars. In our study, the highest values for K, at 52.68 ± 4.21 g kg^−1^, were observed in the 18 h UV-C light group, indicating a remarkably high mineral content compared to literature values. Calcium (Ca) exhibited the highest concentration, reaching 23.24 ± 3.88 g kg^−1^, in the 18 h UV-C light group, in comparison to lettuce varieties like Cogollos de Tudela, Batavia Rubia Munguía, and Maravilla de Verano*,* which reported values ranging from 4.10 to 20.6 mg g^−1^ of dry weight in previous research^42^.

Another mineral of interest in this study was iron, which, according to Liu et al.^43^, is degraded by UV-C light. Under the conditions used in the lettuce samples, the iron contents showed no significant difference between the groups, demonstrating the use of UV-C light does not hinder the absorption of this mineral in the plants. However, the literature reports low (59.90–112.40 μg g^−1^ DW) and high (172–248 μg g^−1^ DW) content values for several popular types of lettuce—the content found in the present study is within such a range^17^. Lettuce cultivation in hydroponics typically involves extended photoperiods, typically ranging from 18 to 20 h of daily light exposure. This practice is driven by several factors that enhance plant growth and yield. Prolonged photoperiods enable continuous photosynthesis, leading to increased energy production and, consequently, greater biomass accumulation. Beyond the enhanced crop yield, this approach offers an ideal environment for vigorous and accelerated vegetative growth. Low intensities and long photoperiods, as carried out in the work, have shown better yields in indoor vegetation cultivation; this is due to the greater photosynthesis produced by prolonged irradiation^33^.

In general, more content of pigments, such as chlorophylls, carotenoids, and phenols, are related to the photosynthetic capacity of the plant to absorb light energy, and even certain limits of long durations of light are beneficial for the plant; in this work, the implementation of the decontamination system in conjunction with a 20 h photoperiod provided better values in pigments and minerals such as Mn and Zn.

## Conclusions

The lettuce production in hydroponics was optimized by reducing the development time with the use of a UV-C decontamination system of water. A reduction in the microbial load optimized the conditions of the final product despite changes in factors such as irradiance in different photoperiods, maintenance of nutritional quality, or, in some cases, its potentiation. Based on the results of the study, it can be concluded that the use of the UV-C decontamination system in hydroponic systems has significant impacts on microbial control, improving the development of vegetables (Supplementary Information). Through experimental observations of faster plant growth, increasing the production of important pigments, and improving antioxidant resistance, without compromising nutrient absorption, we concluded that this improved instrumentation of the hydroponic system would imply an additive to the development of vegetables for food production in controlled environments and for the microbiological safety of crops.

### Supplementary Information

Supplementary Information.

## Data Availability

All data generated and analyzed during this study are included in this published article and its supplementary information files.
